# Characterizing the context of sedentary lifestyles in a representative sample of adults: a cross-sectional study from the physical activity measurement study project

**DOI:** 10.1186/s12889-015-2558-8

**Published:** 2015-12-09

**Authors:** Youngwon Kim, Gregory J. Welk

**Affiliations:** Department of Kinesiology, Iowa State University, Ames, IA USA; MRC Epidemiology Unit, University of Cambridge School of Clinical Medicine, Cambridge, UK

**Keywords:** Sedentary behavior, Epidemiology, Context

## Abstract

**Background:**

Research has clearly demonstrated that excess time spent on sedentary behavior (SB) increases health risks in the population. However, the lack of information on the context of SB in the population prevents a detailed understanding of sedentary lifestyles. The purpose of this study was to characterize the context of SB in a representative sample of adults and to examine differences across various socio-demographic indicators.

**Methods:**

A diverse sample of 1442 adults (ages 20–71 year) completed an interviewer-administered 24-h activity recall to provide detailed information about the time, type and location of the previous day’s activities. All reported activities were matched with MET scores from the Compendium of Physical Activity but only SB (i.e., METS < 1.5) were extracted for the present analyses.

**Results:**

The reported SB were broadly distributed across 5 primary location categories (Work: 27.5 %, Community: 24.8 %, Home/Indoor: 20.5 %, Home/Outdoor: 15.8 %, and Transportation: 11.3 %). Patterns of SB allocations varied considerably across different socio-demographic indicators indicating the extreme variability in SB in the population.

**Conclusions:**

The findings provide unique insights about the context of SB at the population level, and can serve as a guide for developing intervention/policy studies to reduce sedentary time and minimize disparities in SB.

## Background

Evidence indicates that US adults, on average, spend nearly 8 h a day (~55 % of their waking time) being sedentary [1]. Excessive time on sedentary behaviors (SB) is associated with adverse health outcomes, such as mortality rates [2, 3], obesity [4, 5], cardiovascular diseases [6], diabetes [7], and metabolic syndrome [8]. In addition, substantial evidence [9–11] reveals that SB has detrimental effects on various health indicators, irrespective of accumulation of physical activity (PA). Current efforts by public health researchers have focused on ways to assess overall sedentary time (and relations to health outcomes); however, a fundamental gap in knowledge is the lack of understanding of the social and environmental context of SB in the population.

Several seminal studies have emphasized the necessity of understanding specific patterns of SB [12] and PA [13, 14] in order to develop more effective intervention strategies. Behavioral epidemiology frameworks specifically recommend the sequential collection of evidence to optimally inform the development of behavioral interventions [15, 16]. Keys in these models are to better understand the nature and context of the underlying behaviors so that effective interventions can be developed. While considerable research has been performed to understand the context of PA [17–19] relatively little is known about the context of SB. This lack of evidence on the contextual profiles of SB is due, in large part, to the inabilities of traditional measurement tools to assess context of SB (and PA) [20]. Researchers have increasingly relied on objective measurement tools (e.g., accelerometers and inclinometers) to study both PA and SB. While these are useful for capturing time spent in PA and SB, they cannot capture the specific context of behavior (e.g., types and location). Short term recall measures such as the 24 h recall (24PAR) offer considerable potential to study context since it is possible to capture detailed information about individual behavior in a systematic way and with reasonable accuracy [21]. However, to date, there are limited data available to characterize the nature of SB in population-based samples [17].

The present study helps to fill this gap by characterizing the context of SB in a representative sample of adults collected through a large population survey, called the Physical Activity Measurement Survey (PAMS-R01 HL91024-01A1). The PAMS project focused on characterizing PA behavior [22, 23], but the design and measures provide considerable value for also understanding the type, location and context of SB in the population. Therefore, the specific purposes of the study were to characterize the context (type and location) of the most frequently reported SBs and to evaluate the variation according to socio-demographic indicators in this representative sample of adults.

## Methods

### Study design

The PAMS project collected replicate, single-day measures of behavior (using both subjective and objective tools) from a representative sample of over 1400 adults within four ethnically diverse Iowa counties. Participants were contacted through a random digit dialing method (i.e., random selection) with the following inclusion criteria: 20–75 years of age, ability to walk and to perform recall interviews in either English or Spanish. Once recruited, the participants were asked to wear an armband monitor on one randomly selected day and complete a 24PAR interview the following day to recall the specific activities performed on the previous day. The same set of data collection procedures was undertaken on another randomly selected day at least 3 weeks after this first assessment. To facilitate the data collection, staff members made two visits to participants’ homes. Specifically, the first visit occurred before the randomly selected day to obtain informed consent, measure demographic variables, and distribute armband monitors, and the second visit occurred after the randomly selected day to collect the armband monitors used. Data in the overall PAMS project were collected over a 2-year time span to capture the inherent variability in behavioral patterns across seasons. Additional detail on the overall study design, the sampling procedure and the data collection protocol is available [19, 22–24].

### Ethics, consent and permissions

This study was approved by the Institutional Review Board of Iowa State University, and each participant signed informed consent prior to participation. This was done on the initial visit to participants’ homes, which occurred the day before.

### Instrument

The 24PAR is an interviewer-administered measurement tool designed to provide detailed insights about the specific behaviors performed in a 24-h period. The 24PAR has been shown to have high utility and validity, with correlation coefficients > 0.83 (relative to various pattern-recognition technologies) reported from previous validation work [23, 25]. The 24PAR in the present study was administered over the telephone using a Computer Assisted Telephone Interviewing by a trained and supervised data collection team. Respondents were asked to recall the previous day’s activities across four distinct time blocks (i.e., Midnight-6 am, 6 am-Noon, Noon-6 pm and 6 pm-Midnight) in episodes of at least 5 min. Telephone interviewers used the *Blaise* program during the 24PAR interviews in order to identify each reported activity in a given series of activity codes. The appropriate activity would then be chosen from the provided list. Participants were allowed to report a single activity type per time block. Reported activities that could not be located from the list were recorded in text during the interviews, but added to the list as new activity codes after the interviews. The participants provided the purpose and location of all activities performed but only the 5 location codes were used in the present study (Work/Volunteer, Home/indoor, Home/outdoor, Transportation, and Community) codes since the purpose codes captured similar information for SB activities. The interviewers received extensive training prior to the start of the study as well as on-going monitoring by leaders of the team to ensure consistency in data collection procedures among the different interviewers.

### Data reduction

The context and duration of the reported activities on the 24PAR were aggregated by day to create summary files that included a MET score and location code for each reported activity for each participant. The reported minutes were tabulated for each participant to ensure the total accumulated minute equals 1440 (i.e., 60 min × 24 h). For the present report, data were restricted to activities with MET scores ≤ 1.5 (Based on a reduced set of Compendium of PA codes [26]) since this is the established criterion for SB [27]. Activities with assigned MET scores > 1.5 (i.e., light, moderate, and vigorous intensity) were excluded as were periods of reported sleeping and napping. The refined data were merged with participants’ demographic data based on participants’ ID. Socio-demographic variables in the dataset included gender (female and male), age group (20–29 years, 30–39 years, 40–49 years, and 50–71 year), weight status defined by BMI (normal weight, overweight and obese), ethnicity (White, Black, and Other), education background (less than high school, some college/post high school, and college/graduate), and income level (less than $25,000, from $25,000 up to $75,000, and more than $75,000). Data from Trial 2 were not used in the analyses because results were highly similar to those from Trial 1 [19, 23]. Data from the armband monitor were not reported in this study because it does not provide context information of SB.

### Statistical analyses

Time allocations across the five location codes were calculated. The rankings of the 20 frequently reported sedentary activities were determined on the basis of the number of participants that reported the activities at least once in a given day. For each of the top 20 most frequently occurring SBs, average daily reported minutes per person were calculated. Multiple one-way Analyses of Variance (ANOVA) analyses with the Bonferonni adjustment (α = 0.05) were performed to evaluate differences in time allocations between varying levels of each demographic variable across the five location codes. The Jackknife variance estimation method was used to calculate standard errors [28]. Calculated sampling weights were applied to all analyses to account for the complex sampling methodology of the PAMS project. Data management and statistical analyses were performed in STATA/SE Version 12 for Windows (StataCorp LP, College Station, TX).

## Results

A sample of 1648 participants in the PAMS project satisfied the eligibility criteria. However, 1501 participants remained in the final data set since 147 were excluded for the following reasons: choosing not to participate (*n* = 141), death (*n* = 1), relocation (*n* = 3) and/or becoming pregnant (*n* = 2). The sample of 1501 participants produced 2981 data cases. However, 149 observations were deleted for the following reasons: data entry errors with 24PAR (*n* = 18), missing 24PAR data (*n* = 13), outliers on 24PAR (*n* = 7) or problematic data from armband monitors (*n* = 111). The final reduced dataset of 2832 cases was obtained from a unique sample of 1468 participants (1442 from Trial 1 and 1397 from Trial 2), but the analysis for the current study was based on only 1442 participants (from Trial 1). Specific characteristics of the participants are summarized in Table 1. A total of 27 different types of sedentary activities were reported by the participants. The average self-reported daily sedentary time of the total population was 7.7 h. Time spent sedentary increased as the age range increased: 6.7 h for 20–29 years, 7.4 h for 30–39 years, 7.8 h for 40–49 years and 8.0 h for 50–71 year. Females (7.5 h) spent less time being sedentary compared with males (7.8 h).

**Table 1:** Socio-demographic characteristics and reported sedentary time of the participants (*n* = 1442) included

Variables	Values	Sedentary time, hours
Gender		
Female, %	50.6 (1.8)	7.5 (0.2)
Male, %	49.3 (1.8)	7.8 (0.2)
Age (yrs)	46.2 (0.4)	
20–29 years,%	8.2 (1.0)	6.7 (0.5)
30–39 years, %	22.4 (1.7)	7.4 (0.3)
40–49 years, %	34.5 (1.8)	7.7 (0.2)
50–71 year, %	35.0 (1.5)	8.0 (0.1)
Body Mass Index (BMI)	29.9 (0.3)	
Normal Weight, %	25.2 (1.6)	7.7 (0.3)
Overweight, %	32.6 (1.7)	7.7 (0.2)
Obese, %	42.2 (1.8)	7.7 (0.2)
Ethnicity		
White, %	88.6 (1.3)	7.7 (0.1)
Black, %	6.9 (0.9)	8.0 (0.6)
Other, %	4.5 (1.0)	6.9 (0.8)
Education Background		
Less than high school, %	3.1 (0.6)	6.6 (0.7)
High school diploma/some college, %	50.1 (1.8)	7.4 (0.2)
College/graduate school, %	46.8 (1.8)	8.1 (0.2)
Income Level		
Less than $25,000, %	13.5 (1.1)	7.8 (0.3)
From $25,000 up to $75,000, %	43.8 (1.8)	7.2 (0.2)
More than $75,000, %	42.7 (1.9)	8.1 (0.2)

All values were weighted to account for the complex sampling design. Values in parenthesis represent standard errors unless otherwise indicated

**Fig. 1:**
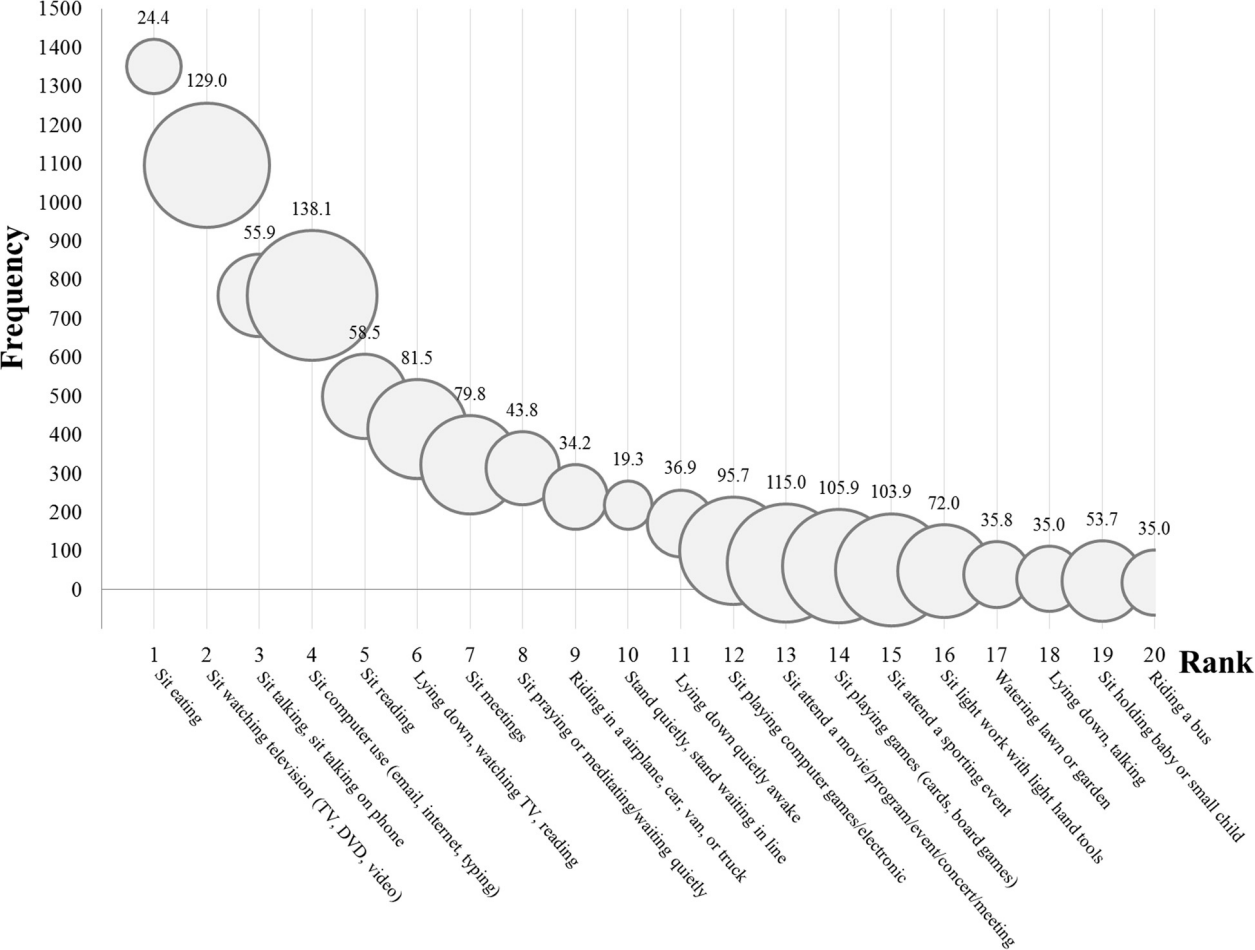
The top 20 most frequently reported sedentary activities. Note: The rank was determined based on the number of participants that reported a sedentary activity at least once in the previous day. The bubble size represents the average daily sedentary time (minutes/day) per activity per person (as shown above the bubbles). All values were weighted to account for the complex sampling design

Figure 1 shows the top 20 most frequently reported sedentary activities along with corresponding average daily sedentary minutes per activity per person; the bubble sizes are proportional to the reported sedentary minutes (as shown above the bubbles). The most commonly reported sedentary activity was ‘sit eating’ (*n* = 1351), followed by ‘sit watching television’ (*n* = 1096), ‘sit talking on phone’ (*n* = 760) and ‘sit computer use’ (*n* = 760). Of the 20 sedentary activities, 15 (including ‘riding in a airplane, car, van, or truck’ and ‘riding a bus’) involved a form of ‘sitting’ in the definition. When ranked by the volume of sedentary time, the top three activities were ‘sit computer use’ (138.1 min/day), ‘sit watching television’ (129.0 min/day), and ‘sit attend a movie/program/event/concert/meeting’ (115.0 min/day). The top 3 sedentary activities by location categories were: ‘sit eating’ (*n* = 356), ‘sit computer use’ (*n* = 335) and ‘sit talking on phone’ (*n* = 185) for Work, ‘sit eating’ (*n* = 1195), ‘sit watching TV/DVD/Video’ (*n* = 1072), and ‘sit computer use’ (*n* = 519) for Home/Indoor, ‘sit talking on phone’ (*n* = 28), ‘watering lawn or garden’ (*n* = 24), and ‘sit praying or meditating/waiting quietly’ (*n* = 20) for Home/Outdoor, ‘riding in airplane/car/van/truck’ (*n* = 231), ‘riding a bus’ (*n* = 19) and ‘sit talking on phone’ (*n* = 8) for Transportation, and ‘sit eating’ (*n* = 337), ‘sit talking on phone’ (*n* = 293) and ‘sit praying or meditating/waiting quietly’ (*n* = 177) for Community.

**Fig. 2:**
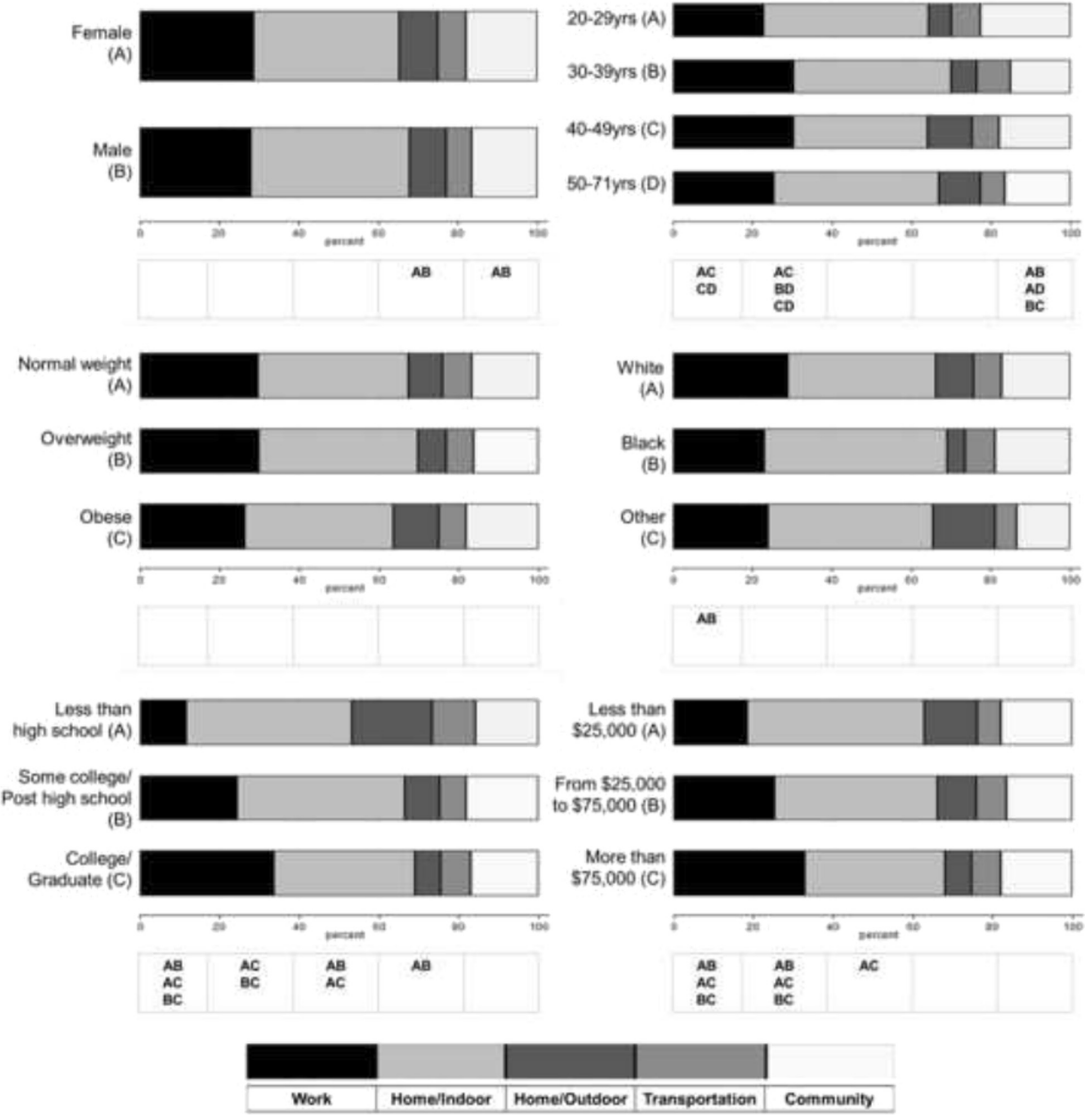
Time allocations of five location codes (Work, Home/Indoor, Home/Outdoor, Transportation, and Community) across six socio-demographic variables. Note: Significant differences are indicated by combinations of ‘A’, ‘B’, and ‘C’. All values were weighted to account for the complex sampling design

In regards to the location categories, the overall time allocations were 27.5 % for Work, 24.8 % for Community, 20.5 % for Home/Indoor, 15.8 % for Home/Outdoor, and 11.3 % for Transportation. Figure 2 specifically shows time allocations of the 5 location categories by 6 different socio-demographic variables. Females reported significantly greater time spent being sedentary during transportation (*P* = 0.046) and in the community (*P* = 0.021) than males. Older individuals (ages 50–71 year) reported significantly greater sedentary time at Home/Indoor (*P* values ranging from <0.001 to 0.003), but less sedentary time at Work (*P* = 0.006) and Community (*P* = 0.035), compared with younger individuals. White people reported a significantly larger time allocation at Work (*P* = 0.003) compared with Black people. Individuals with relatively higher education levels exhibited significantly larger sedentary time spent at Work (*P*s < 0.001), but smaller sedentary time at Home/Indoor and Outdoor (*P*s ranging from <0.001 to 0.003), compared with those with lower education levels. Individuals with higher income levels reported significantly larger sedentary time spent at Work (*P*s < 0.001), but less sedentary time at Home/Indoor (*P*s < 0.001) and Outdoor (*P* = 0.035), in comparison with individuals with lower income levels.

## Discussion

There has been considerable interest in understanding the specific context in which SB and PA occur [13, 14]. Research on this topic, however, has been limited by the inability of traditional measurement tools (i.e., accelerometers, long-term recall methods) to capture contextual information. The PAMS study was designed specifically to address this gap and to improve the utility of self-report data for public health research [22]. The established 24PAR protocol [23, 29] was refined to capture contextual variables, which enabled us to obtain detailed insights about the specific types and location of SB and its disparities by socio-demographic status in a representative sample of adults.

A previous study by Tudor-Locke et al. [17] that examined SB (and PA) patterns from the American Time Use Survey (ATUS) reported similar rankings of predominant sedentary activities in a large population sample. For example, they reported the same general rankings for eating (Rank 1), TV viewing (Rank 2), and other sedentary activities (i.e., cell-phone use, talking, transportation sedentary activities, etc.) as reported herein. The ATUS used a similar type of 24PAR (and the activities were also coded with the same Compendium codes), but a unique advantage of the present study is that we were able to provide insights about the location information of the reported activities. The patterns were somewhat different than those reported in Keadle et al. study [30] utilizing the traditional computerized version of the 24PAR [25]. For example, the Keadle et al. study [30] found the Work/School category (130.7 min) accounted for most of the total reported sedentary time, with substantially smaller sedentary minutes spent at Home (59.8 min) and in the Community (39.5 min). In the current study, we found 36 % of total sedentary time to be explained by the Home category, followed by 27.5 % at Work, 24.8 % in the Community and 11.3 % during Transportation. The differential patterns of time allocations between this study and the Keadle et al. [30] study may be attributable to the difference in the types of location categories. However, the major difference is likely that Keadle et al. [30] used a convenient sample of 15 adults (aged 18–75years) whereas the current study utilized a representative sample of over 1400 adults that were randomly selected through a multi-stage sampling procedure. The Keadle et al. study [30] provided evidence to support the validity of the reported 24PAR location (and purpose) codes so it was not designed with the same goal as the PAMS project.

A unique advantage of the present study is that it also demonstrates that individuals with different levels of socio-demographic indicators exhibited differential patterns of SB as well as different contextual explanations for where (i.e., location) the participants spent time sedentary. The findings, in general, yielded intuitive findings for comparisons across the 6 socio-demographic variables but some examples are noteworthy. For example, younger individuals (i.e., 20–29 years) reported less sedentary time at ‘work’, but more sedentary time at ‘Home/Indoor’ compared with older individuals (40–49 years). This may be because younger individuals may be less likely to be employed at sedentary jobs (compared with older adults). The larger allocation for the Home/Indoor category may reflect younger individuals’ greater interest in popular sedentary activities such as playing video games, using computers and/or watching TV, all of which are likely to occur at home. Similar comparisons were observed for the other socio-demographic indicators (i.e., Ethnicity, Education and Income). For example, individuals that are white or with higher levels of academic background (i.e., college graduates) and/or income (i.e., from $75,000 up to $100,000) reported being more sedentary‘at work’ and less sedentary at home (compared with those that are black or with lower levels of academic background and/or income). These findings clearly suggest that the appreciation of the specific context is critical for understanding the complex behavioral aspects of SB at the population level.

A few intervention studies have adopted context-specific approaches in order to reduce sedentary time and mitigate disparities of SB across various adult populations. For example, Schuna et al. [31] found that the intervention group that used treadmill desks for 3 months at a workplace led to reductions in sedentary time (in addition to increased physical activity) compared with the control group that worked in usual working conditions. Another intervention study by Lakerveld et al. [32] reported no significant differences in sedentary time between the intervention group (that received counseling about adopting healthy lifestyles) and control group (that received only brochures) at the 6-, 12- and 24-month follow-up. However, they found variation of intervention effects by academic background in the intervention group: for example, increases in sedentary time for individuals that finished secondary school. Rosenberg et al. [33] found significant decreases in sitting time and increases in sit-to-stand transitions as assessed with the activPal, and significant decreases in overall sedentary time as well as increases in physical activity time as assessed with the Actigraph in an 8-week theory-based intervention with 25 overweight/obese older adults (mean age of 71.4 years). While these intervention studies suggest the effectiveness of context-specific intervention approaches in reducing sedentary time, additional research is needed to test these effects with longer follow-up terms and in a larger sample of adults.

The current study is not without limitations. We used only a MET-derived criterion (≤1.5MET) to define SB, which is the same methodology used in the previous study by Tudor-Locke et al. [17]. However, the definition of SB suggested by the Sedentary Behaviour Research Network [34] incorporates a posture component (i.e., sitting or reclining) in addition to the energy expenditure component (i.e., ≤1.5MET). By this definition, 1 of the top 20 sedentary activities (‘Stand quietly, stand waiting in line’) would not have been classified as a SB. Applying the same principle, 4 of the top 20 light (i.e., >1.5MET) activities (e.g., ‘driving light truck’ [*n* = 1191], ‘sit deskwork/paperwork/writing’ [*n* = 281], ‘sit child care’ [*n* = 158] and ‘sit play with child’ [*n* = 132]) would have been categorized as sedentary activities. Another limitation is that the results from this study might not be generalizable to the entire US adult population, given that the participants were recruited from a single state of the US. However, the reported daily sedentary time (i.e., 7.7 h/day) matched the national average of sedentary time (estimated by an objective monitor) in US adults [1]. This may suggest that our sample shares similar characteristics with the whole US adult population. Moreover, the telephone-administered 24PAR used herein has not been directly validated for assessing context of SB. However, the previous validation study [23] found it to have acceptable agreement relative to an armband monitor in estimating energy expenditure. Moreover, context information captured by a computerized-24PAR (analogous to the telephone-based 24PAR) was relatively comparable with context information measured by direct observation [30].

## Conclusions

The present study provided comprehensive context information of SB (i.e., types and location) to advance understanding about SB in adults. The findings provide breakdowns of the time spent in different categories but the more notable finding is the diverse range in profiles across the various socio-demographic groups. These findings may have value for understanding disparities of SB and health in the population. Evidence from this study can serve as a fundamental framework for designing and implementing future intervention studies aimed at reducing sedentary time at the population level.
